# Testing for amphetamine-type stimulant (ATS) use to ascertain validity of self-reported ATS use among young female sex workers in Cambodia

**DOI:** 10.1186/1940-0640-7-11

**Published:** 2012-06-28

**Authors:** Vannda Kab, Jennifer Evans, Neth Sansothy, Ellen Stein, Marie Claude-Couture, Lisa Maher, Kimberly Page

**Affiliations:** 1 School of Public Health, University of California at Berkeley, Berkeley, California, USA; 2 Department of Epidemiology and Biostatistics, University of California San Francisco, 50 Beale Street, Ste 1200, San Francisco, CA, 94105, USA; 3 Cambodia National Center for HIV, AIDS, Dermatology and STDs, Phnom Penh, Cambodia; 4 The Kirby Institute (formerly the National Centre in HIV Epidemiology and Clinical Research), Sydney, New South Wales, Australia

## Abstract

**Objective:**

To assess concordance between self-reported amphetamine-type stimulant (ATS) use and toxicology results among young female sex workers (FSW) in Phnom Penh, Cambodia.

**Methods:**

Cross-sectional data from the Young Women’s Health Study-2 (YWHS-2), a prospective study of HIV and ATS use among young (15 to 29 years) FSW in Phnom Penh, Cambodia, was analyzed. The YWHS-2 assessed sociodemographic characteristics, HIV serology, HIV risk, and ATS use by self-report and urine toxicology testing at each quarterly visit, the second of which provided data for this assessment. Outcomes include sensitivity, specificity, positive- and negative predictive values (overall and stratified by age), sex-work setting, and HIV status.

**Results:**

Among 200 women, prevalence of positive toxicology screening for ATS use was 14% (95% confidence interval [CI], 9.2, 18.9%) and concurrent prevalence of self-reported ATS was 15.5% (95% CI, 10.4, 20.6%). The sensitivity and specificity of self-reported ATS use compared to positive toxicology test results was 89.3% (25/28), and 96.5% (166/172), respectively. The positive predictive value of self-reported ATS use was 80.6% (25/31); the negative predictive value was 98.2% (166/169). Some differences in concordance between self-report and urine toxicology results were noted in analyses stratified by age group and sex-work setting but not by HIV status.

**Conclusion:**

Results indicate a high prevalence of ATS use among FSW in Phnom Penh, Cambodia, and high concordance between self-reported and toxicology-test confirmed ATS use.

## Introduction

Cambodia has the highest HIV prevalence of any Asian country, and over the last decade has experienced the most serious HIV/AIDS epidemic in Southeast Asia [1]. Heterosexual contact is the major route of HIV transmission, and female sex workers (FSW) remain the group at highest risk [2]. Although crucial progress has been made in reducing risky sexual behavior, including widespread condom use and promotion of reduced number of sexual partners, HIV prevalence among FSW remains high, ranging from 11% to 26% [2-5]. Poverty [6], low literacy [7], a high prevalence of sexually transmitted infections (STI) [4], and a highly mobile workforce [8] are contributing factors to the epidemic. Recent research has also identified drug use and, in particular, amphetamine-type stimulant (ATS) use as a serious emerging problem associated with HIV risk among FSW [3,9,10], which threatens to reverse downward trends in HIV infection rates in the region.

Amphetamine-type stimulants include a range of synthetic psychostimulants, including methamphetamine, amphetamine, and ecstasy, which can be injected, smoked, or taken orally. Effects of these drugs include euphoria, alertness, arousal, increased libido, increased sympathetic nervous responses, (heart rate, respiratory rate, blood pressure), and perceived increases in confidence, energy and physical strength [11]. In Cambodia, a pill form of methamphetamine known as “yama” is widely produced, trafficked, and used. The tablets generally contain about 25% methamphetamine. “Crystal” (also known as “ice”) is generally about 85% methamphetamine and more addictive. Although yama pills are swallowed, both forms are usually melted and the vapors inhaled, resulting in rapid neurologic effects [11,12]. Use of ATS has been associated with elevated HIV risk behavior in many countries and in several population subgroups [3,9,13-19]. The United Nations Office on Drugs and Crime (UNODC) reports that use of these drugs is widespread in Asia and increasing rapidly in Cambodia [20]. In Cambodia, ATS are highly available both in pill and crystalline form and are generally ingested or smoked; injection use is uncommon [21]. The Cambodia National Authority for Combating Drug Abuse (NACD) estimated that 70% of all drug users in Cambodia use ATS [20]. The drug accounts for the majority of all drug seizures by authorities, and, in pill form, has been ranked as the leading drug of abuse for the past nine years with consistent increases since 2006 [20], at which time it was estimated that 30,000 tablets of yama were consumed orally or smoked there daily. Use is particularly high among vulnerable populations, including FSW [3,9], men who have sex with men (MSM), and street children [12,20,21].

Measuring drug use in epidemiological research studies poses challenges. Self-reported measures of drug use have the advantage of being noninvasive and permit evaluation over longer time periods compared with biochemical assessments [22]. However, study participants may misrepresent drug use due to social desirability bias, stigma, poor recall, poorly worded questions, or poorly worded response categories in surveys and interviews [23,24], all of which could result in misclassification of measured exposures. Although studies have shown that the use of Audio Computer-Assisted Self Interview (ACASI) increases reporting of sensitive and stigmatized behaviors [25,26], research suggests that the validity of self-reported drug use varies by population [27], race/ethnicity [28-30], mental health [27], and drug treatment status [22,31,32], although not by gender [30,33]. Accuracy has varied in studies of arrestee populations [34,35] but have been reported as higher in groups sampled in emergency department and STI clinics [34]. Those that report more frequent drug use, compared to infrequent use, are more likely to self-report recent drug use [27,36]. Urine toxicology assessments provide sensitive and valid measures of many drug types; but some, like ATS, are restricted to a short timeframe due to rapid metabolization. The detection window may also depend on the physical condition of the individual (e.g., degree of hydration), route of drug ingestion (e.g., oral, intranasal, or intravenous), frequency of use, and drug-related factors such as purity [27].

To explore the validity of self-reported ATS use among young FSW in Phnom Penh, Cambodia, we compared self-reported ATS use with results from concurrently collected urine toxicology tests. We also examine whether sociodemographic, sex-work venue, and HIV status were associated with validity of self-reported ATS use.

## Methods

### Study setting

The Young Women’s Health Study (YWHS-2) was a prospective study of HIV and ATS use among 15–29 year-old women engaged in sex work in a diversity of settings in Phnom Penh, Cambodia [10]. Data for this cross-sectional validity assessment was collected at the second quarterly study visit women attended. Both self-reported measures and urine toxicology testing for ATS were assessed. The YWHS-2 was led by HIV-research and HIV-prevention specialists from the Cambodian National Center for HIV, AIDS, Dermatology, and STDs (NCHADS), the Cambodian Women’s Development Association (CWDA), the University of California San Francisco (UCSF) in the United States, and the Kirby Center (formerly National Centre for HIV Epidemiology and Clinical Research) at the University of New South Wales (UNSW) in Australia.

### Study participants and data collection

Young women at high risk of HIV infection were the target study population. Inclusion criteria were age 15–29 years, understanding of spoken Khmer, Cambodian ethnicity, reporting of at least two different sexual partners in the last month *or* engaging in transactional sex (sex in exchange for money, goods, services, or drugs) within the last three months, plans to stay in the Phnom Penh area for 12 months, being biologically female, and being able to provide voluntary informed consent. Study methods have been described previously [3]. In brief, trained field assistants from the CWDA recruited women from community locations, provided study information, and obtained group informed consent. Women who consented were then seen by appointment at the YWHS-2 clinic site; free transportation was provided. Participants received US $5 and condoms at each study visit. Contact information was collected to facilitate participant tracking and maximize follow-up. Women were asked to enroll for a one-year study with quarterly study visits.

Data collection for this validity assessment occurred in November 2009 at the second study visit (month 3 of the study). All study visits included administration of a structured questionnaire in Khmer by trained interviewers who queried participants about sociodemographic characteristics, health care, occupational and sexual risk exposures, alcohol and self-reported ATS use as well as testing for HIV and ATS using blood and urine samples, respectively. The second visit (at 3 months) was used for this cross-sectional analysis because questions about past 48-hour drug use (corresponding to when urine toxicology screening could detect ATS) were added to the questionnaire starting with that visit; thus, it was the first available time point for comparison and validation of self-reported use and urine testing. Women were asked about type of ATS use (yama, ice, and/or crystal); frequency and route of use (ingestion, smoking or injected) since their last (baseline) visit; number of days of use in the past month; and use in the past five days, with specific questions including “today,” “yesterday,” and “two,” “three,” “four,” and “five” days ago. Urine toxicology testing (Innovacon Multi-Drug Screen Test Panel Dip, Redwood Toxicology Laboratories, Santa Rosa, CA) was conducted to qualitatively screen for recent ATS, opiate, and cannabis use. Women were asked to void into prelabeled sterile collection cups in a private lavatory; the specimens were passed through a private window to the on-site laboratory for testing. The test included four strips, which yielded positive results for amphetamine and/or methamphetamine if either exceeded 1000 ng/mL; for opiates if morphine in urine exceeded 2000 ng/mL; and for cannabis if the concentration 11-nor-Δ9-tetrahydrocannabinol-9-carboxylic acid (THC-COOH) exceeded 50 ng/mL. A positive amphetamine or methamphetamine screen (the primary outcome of interest) was considered indicative of ATS use in the past 48 hours. Drug screening was conducted in conjunction with client-centered risk reduction counseling.

### Ethical review

The study protocol was reviewed and approved by the Cambodian National Ethics Committee, the University of California San Francisco Institutional Review Board, and the University of New South Wales Human Research Ethics Committee.

### Data analysis

The primary outcomes of interest were ATS use by self-report in the past two days and ATS use by urine screening. Correlates of interest included age, HIV status, and sex-work venue. Both self-report and urine-screened ATS coded as dichotomous variables. Descriptive statistics including measures of central means, medians and ranges, and frequencies were assessed for continuous and categorical variables, respectively. Chi-square tests were used to assess differences between proportions, including self-reported past two-day use of ATS and urine toxicology ATS results. Sensitivity, specificity, positive- and negative-predictive values, and respective 95% confidence intervals (95% CI) were computed. We conducted sub-analyses stratifying by age, HIV status, and sex work setting. Analyses were performed using STATA/IC 11.1 (STATA, College Station, TX, USA.)

## Results

Two hundred twenty women were enrolled in YWHS-2. Two hundred (91%) returned for the second visit and were included in this analysis. The median age of participants was 26 (Interquartile range (IQR), 22–28 years). A quarter (24%) had no education (median, 4 years [IQR 1–6 years]), and less than half (42.5%) were married or cohabitating with a partner. Median number of years working in transactional sex was 3.6 years (IQR, 2–6 years); most (74%) currently worked in entertainment and service settings. The median number of sex partners in the past 30 days reported was 4 (IQR, 2–10.5 partners) (Table 1).

**Table 1:** Selected sociodemographic and sexual risk characteristics of women participating in the Young Women’s Health Study-2

**Characteristic**	**n**	**%**
**Age**		
16-18	11	5.5
19-24	62	31.0
25-29	127	63.5
**Years of schooling (0-12 years)**		
No Education	48	24.0
Primary (1-6 years)	114	57.0
Secondary (≥ 7 years)	38	19.0
**Marital status**		
Married/Living together	85	42.5
Divorced/separated/widowed		
Single	34	17.0
**Years in transactional sex**		
≤ 5 years	138	69.0
6-10 years	53	26.5
≥ 10 years	9	4.5
**Type of sex venue**		
Entertainment/other	147	73.5
Brothels/freelance	53	26.5
**Number of sex partners within last 30 days**		
≤ 10 partners	150	75.0
11-29 partners	48	24.0
≥ 30 partners	2	1.0

All participants provided urine samples for ATS testing; 14% (95% CI, 9.2, 18.9) were positive, indicating use in the past 48 hours. The prevalence of self-reported ATS use for the same detection period was 15.5% (95% CI, 10.4, 20.6).

### Sensitivity of self-report

The sensitivity of self-report was high: 89.3% (95% CI, 77.8, 100) of women who screened positive on the urine toxicology reported recent use of ATS in the same detection period (Table 2). Sensitivity of self-reported ATS use was lower (71.4%) in women aged 19–24 years compared with the other two groups (100% in women under 19 years and 95% in women over 24 years). Women who worked in the entertainment/service sector had a lower sensitivity of self-reported ATS use (60%) than those working freelance and/or in brothels (95.7%) (Table 3c). Sensitivity did not vary by HIV status (Table 3).

**Table 2:** Self-reported versus urine-toxicology-detected amphetamine-type stimulant use among women participating in the Young Women’s Health Study-2


		**Urine toxicology result (n)**	
		**Positive**	**Negative**
Self-reported use	Yes	25	6
	No	3	166
	**Percentage**		**95% CI**
Sensitivity	89.3		77.8–100
Specificity	96.5		93.8–99.2
Positive predictive value	80.6		66.7–94.5
Negative predictive value	98.2		96.2–100

**Table 3:** Self-reported amphetamine-type stimulant use compared with urine toxicology results by age, HIV status, and sex-work venue


**Self-reported ATS use compared to urine toxicology results by age group**			
**Age 16-18 years**		**Urine toxicology results (n)**	
		Positive	Negative
Self-reported use (n)	Yes	2	1
	No	0	8
			**95% CI**
Sensitivity (%)	100		−−
Specificity (%)	88.9		68.4–100
Positive predictive value (%)	66.7		13.3–100
Negative predictive value (%)	100		−−
**Age 19-24 years**		**Urine toxicology results (n)**	
		Positive	Negative
Self-reported use (n)	Yes	5	3
	No	2	52
			**95% CI**
Sensitivity (%)	71.4		38.3–100
Specificity (%)	94.6		88.5–100
Positive predictive value (%)	62.5		29.0–96.0
Negative predictive value (%)	96.3		91.3–100
**Age 25-29 years**		**Urine toxicology results (n)**	
		Positive	Negative
Self-reported use (n)	Yes	18	2
	No	1	106
			**95% CI**
Sensitivity (%)	94.7		84.7–100
Specificity (%)	98.2		95.6–100
Positive predictive value (%)	90.0		76.9–100
Negative predictive value (%)	99.1		97.2–100
**Self-reported ATS use compared to urine toxicology results by HIV status**			
**HIV positive**		**Urine toxicology results (n)**	
		Positive	Negative
Self-reported use (n)	Yes	9	2
	No	1	21
			**95% CI**
Sensitivity (%)	90.0		71.4–100
Specificity (%)	91.3		79.8–100
Positive predictive value (%)	81.8		59.0–100
Negative predictive value (%)	95.5		86.8–100
**HIV negative**		**Urine toxicology results (n)**	
		Positive	Negative
Self-reported use (n)	Yes	16	4
	No	2	145
			**95% CI**
Sensitivity (%)	88.9		74.4–100
Specificity (%)	97.3		94.7–99.9
Positive predictive value (%)	80.0		62.5–97.5
Negative predictive value (%)	98.6		96.8–100
**Self-reported ATS use compared to urine toxicology results by type of sex work setting**			
**Entertainment and/or service**		**Urine toxicology results (n)**	
		Positive	Negative
Self-reported use (n)	Yes	3	3
	No	2	131
			**95% CI**
Sensitivity (%)	60.0		17.1–100
Specificity (%)	97.8		95.3–100
Positive predictive value (%)	50.0		10.0–90.0
Negative predictive value (%)	98.5		96.4–100
**Freelance and/or brothel**		**Urine toxicology results (n)**	
		Positive	Negative
Self-reported use (n)	Yes	22	3
	No	1	35
			**95% CI**
Sensitivity (%)	95.7		87.3–100
Specificity (%)	92.1		82.5–100
Positive predictive value (%)	88.0		75.2–100
Negative predictive value (%)	97.2		91.9–100

### Specificity of self-report

Specificity of self-report was high overall: 96.5% (95% CI, 93.8, 99.2) of women who had negative ATS urinalysis reported no ATS use (Table 2). Table 3 shows measures of specificity by age, HIV status, and sex-work setting. Specificity of self-report was lowest among very young women (<19 years) (88.9%) compared with women in the older age groups (94.6% and 98.2% among women age 19–24 years and 25–29 years, respectively); among HIV-positive women (91.3%) compared with HIV-negative women (97.3%); and among women working freelance and/or in brothels (92.1%) compared with women working in the entertainment sector (97.8%).

### Positive predictive value of self-report

Among 31 women who reported using ATS use within the last 48 hours, 80.6% had positive urine toxicology tests (95% CI, 66.7, 94.5). When analyzed by age group, the youngest groups (ages <19 years and 19–24 years) had lower positive predictive value (PPV) (62.5 and 66.7%, respectively) compared with the oldest age group (90%). Women who worked in the entertainment sector had lower PPV (50%) of self-report compared with those who worked in freelance or brothel settings (88%). Participants’ HIV status did not appear to affect PPV (Table 3).

### Negative predictive value of self-report

Overall, the negative predictive value (NPV) of self-reported ATS use was high: 98.2% (95% CI, 96.2, 100) of women who reported no ATS use in the last 48 hours had negative urine toxicology tests (Table 2). These results were consistent by age group, HIV status, and sex work venue (Table 3).

## Discussion

Overall, results suggest high validity of self-reported ATS use among FSW when compared with urine toxicology screening. In almost all cases (98.2%) where women reported no ATS use in the past two days, negative urinalysis corroborated self-report. The majority of participants (89.3%) with positive urine tests reported ATS use during the same detection period. However, only 81% of participants (25/31) who reported ATS use had positive urine tests. One possible explanation of the low positive predictive value is that women in the study actually used ATS but in such a small quantity that the urine tests failed to detect it. Since ATS is illegal and its purity is unknown, some women could have used the less pure forms of ATS, which may not have been potent enough to be detected by urine testing. The NACD has reported that, among 151 pill samples of ATS tested, 25% of the samples had purities below 10% [21]. Although the proportion of women self-reporting ATS use was slightly higher (15.5%) than the urine test results (14%), these rates are not inconsistent and are near perfect. Other studies have documented higher self-reported use compared with urinalysis results, leading to recommendations that multiple methods be used to assess drug use exposures [27,37,38]. The high concordance between self-report and test results are suggestive of high internal validity of self-report of ATS in our study population.

Some differences were seen in the performance of self-report compared with urinalysis when examined by age, HIV status, and sex-work setting. Most notably, there was lower precision between positive self-report and urinalysis tests among younger women and among women working in entertainment or service settings. The lower PPV may relate to lower prevalence of ATS use among these subgroups. We have previously shown that women working in entertainment and service sectors in Cambodia are less likely to use ATS than women working in brothels (17.7% compared with 35.6%) [9]. Prevalence of ATS among younger women is slightly lower but not significantly so [9]. Importantly, specificity was high overall, with subgroup analyses showing valid self-report of no ATS use in our sample. This is important for further studies of ATS exposure in this population, for public health surveillance, and potentially for intervention and implementation of drug prevention programs.

The high validity of self-report may be associated with several factors. The women in this study were not reluctant to answer the survey questions or to take the test, as indicated by the high participation rate. This could be due, at least in part, to the fact that the participants were recruited by a known and trusted community-based agent, our collaborating partner (the CWDA), and were comfortable with the staff involved in data collection. Moreover, the women in the study knew that providing truthful responses about their drug use would not result in negative consequences or punitive action.

This study had several limitations. Due to the small sample size and nonsystematic sampling, our estimates lack precision and results may not be representative of all young women engaged in sex work in Phnom Penh or Cambodia. This is particularly true for the stratified analyses, where cell sizes were very small in some cases and prevalence of ATS was lower. Poor recall may have contributed to some discordance, including the relatively low PPV found overall. Approximately one in five women (19%) incorrectly reported recent ATS use. Recall of ATS use could be affected by recent ATS use and its side effects, including sleep deprivation and confusion. It is unknown if this would result in over- or underestimating of self-report. Since women were all informed about the testing as part of the informed consent process and ongoing study-procedure education, some women may have over-reported use for the periods about which they were queried. Moreover, urine toxicology tests are not perfectly accurate [35]. Although the urinalysis test is widely accepted as a “gold standard” for substance use validation [39], exclusive reliance on such results does not necessarily improve validity because of problems with false negatives [40]. Many studies comparing self-report, urine, and hair testing results suggest that hair analyses provide higher rates of recent drug use than can be detected by either urine tests or self-reports [27]. Various authors suggest multi-modal testing for the most accurate results [27,38,41].

Despite these limitations, our results suggest a high level of concordance between self-reported ATS use and urine toxicology results in this group of women. Results indicate high prevalence (14%) of ATS use among FSW, who are also at elevated risk of HIV and other sexually transmitted infections [3,9]. There are few, if any, community-based options for ATS users in Cambodia. The finding that self-report, especially specificity, is valid among young FSW is important because of potential utility in surveillance as well as drug prevention and intervention programs in this population. There is a significant need for evidence-based prevention and drug treatment resources in Cambodia, including potentially cognitive behavioral therapy, contingency management, and possibly new pharmacotherapies to reduce ATS use [42-47]. The forthright self-reporting of drug use by women participating in this study shows that, in a safe and nonpunitive setting, disclosure of accurate drug use is possible. These findings, which are consistent with other studies showing high validity of self-reported drug use, may also be relevant to other vulnerable populations in Cambodia reported to have high rates of ATS use and who may also be in need of interventions, including children, young adults, and men who have sex with men [20]. Indeed, with escalating manufacture and use of ATS throughout Southeast and East Asia, and in consideration of the need for expanded surveillance of drug use to more accurately inform public health and policy responses, self-reported use may be a reliable data collection method. For surveillance, research, and health-care settings, it is important that providers and others address drug-related health issues in a nondiscriminatory manner and without punitive consequences in order to accurately assess and effectively address health and safety issues in high-risk populations.

## **Competing interests**

The authors declare that they have no competing interests.

## **Authors' contributions**

All authors contributed to this study: VK, participated in the study design, the primary data analysis and interpretation, and drafted the manuscript; JE contributed to the study conception and design, statistical analysis and interpretation, drafting and review of the manuscript; NS, ES, and M-CC, contributed to the conception and implementation of the study, participated in data collection, and reviewed and provided feedback on the manuscript; LM contributed to the conception and design of the study, and reviewed and edited the manuscript; KP conceived of the study, oversaw data collection and implementation, reviewed and edited the final manuscript. All authors read and approved the final manuscript.
